# Inclusion of Vitamin A Intake Data Provides Improved Compartmental Model-Derived Estimates of Vitamin A Total Body Stores and Disposal Rate in Older Adults

**DOI:** 10.1093/jn/nxz056

**Published:** 2019-05-16

**Authors:** Michael H Green, Jennifer Lynn Ford, Joanne Balmer Green

**Affiliations:** Department of Nutritional Sciences, College of Health and Human Development, The Pennsylvania State University, University Park, PA

**Keywords:** humans, model-based compartmental analysis, retinol, stable isotopes, tracer kinetics, vitamin A stores, WinSAAM

## Abstract

**Background:**

Sampling times and study duration impact estimates of kinetic parameters and variables including total body stores (TBS) and disposal rate (DR) when compartmental analysis is used to analyze vitamin A kinetic data.

**Objective:**

We hypothesized that inclusion of dietary intake (DI) of vitamin A as an additional input would improve confidence in predictions of TBS and DR when modeling results appear to indicate that studies are not long enough to accurately define the terminal slope of the plasma retinol isotope response curve.

**Methods:**

We reanalyzed previously published data on vitamin A kinetics monitored over 52 d in 7 US and 6 Chinese adults (means: 56 y, BMI 26.6 kg/m^2^, 38% males), adding an estimate for vitamin A intake [2.8 µmol/d (mean RDA)] as an input during application of the Simulation, Analysis and Modeling software.

**Results:**

Use of a model with 1 extravascular compartment (1 EV), as in the original analysis, resulted in predictions of vitamin A intake that were higher than physiologically reasonable; inclusion of intake data in a model with 2 extravascular compartments (2 EV DI) resulted in more realistic estimates of intake and DR. Specifically, predictions of DR by the 2 EV DI (versus 1 EV) model were 2.10 compared with 12.2 µmol/d (US) and 2.21 compared with 5.13 µmol/d (Chinese). Predictions of both TBS [2056 compared with 783 µmol (US) and 594 compared with 219 µmol (Chinese)] and days of vitamin A stores [981 compared with 64 d (US) and 269 compared with 43 d (Chinese)] were higher using the new approach.

**Conclusions:**

Inclusion of vitamin A intake as additional data input when modeling vitamin A kinetics can compensate for less-than-optimal study duration, providing more realistic predictions of vitamin A TBS and DR. This approach advances the application of compartmental analysis to the study of vitamin A and, potentially, other nutrients.

## Introduction

Model-based compartmental analysis (1, 2) has been used to describe and quantitate whole-body vitamin A metabolism (3) in rats [e.g., see (4–7)], monkeys (8), and humans (9–13). By applying this approach to data on plasma retinol kinetics after ingestion of labeled preformed vitamin A by humans, modeling has been used to estimate vitamin A pool sizes, and it thus complements isotope dilution and other tools as an independent method for assessing vitamin A status. As shown in the first vitamin A compartmental modeling study done in rats (14), and in later experiments in humans (9, 11, 13, 15), it is crucial to obtain data that adequately define the system kinetics so that model predictions of vitamin A stores and other parameters are accurate. Thus, study duration and sampling schedules, among other factors, are important considerations in designing experiments to which compartmental modeling will be applied (16). On the other hand, these need to be viewed in light of practical and ethical issues.

When Cifelli et al. (10) applied compartmental analysis for the first time to retinol kinetic data obtained in human studies that had been designed for modeling (17, 18), a duration of 52 d was deemed long enough to adequately define the system kinetics. Data for well-nourished older adults were fit to a 6-compartment model in which plasma retinol exchanged with vitamin A in 1 extravascular pool that includes vitamin A stores. Model predictions of vitamin A stores [892 ± 637 µmol (mean ± SD) for 12 US subjects and 233 ± 109 µmol for 14 Chinese subjects] were similar to those estimated by Furr et al. (19) after liver biopsy in 10 US subjects (838 ± 628 µmol). Predictions of vitamin A disposal rate (DR) in the study by Cifelli et al. (10) were 15 ± 6 µmol/d for the US subjects and 6 ± 2 µmol/d for the Chinese. Because modeling was done under the assumptions of a steady state (in which functional input equals output) and a fixed vitamin A absorption efficiency of 75%, a DR of 15 µmol/d translates to a vitamin A intake of 20 µmol/d or >5000 µg retinol activity equivalents/d for the US subjects. This intake seems high in light of the mean RDA for adults (2.8 µmol/d or 800 µg retinol activity equivalents/d). In addition, considering the age and health status of these individuals, predictions of “days of vitamin A stores” were quite low (59 ± 28 d for US subjects and 43 ± 15 d for Chinese), especially for the US subjects whose vitamin A stores were relatively high. Although these points were not specifically addressed by Cifelli et al. (10), in retrospect, they merit attention.

Although the study length used in the experiments by Tang et al. (17) and Wang et al. (18) that generated the data later analyzed by Cifelli et al. (10) was selected with modeling in mind, it is now evident that a longer experiment would have better defined the system kinetics (especially the terminal slope of the plasma isotope response curve) and thus provided different values for vitamin A intake and DR [and consequently different predictions of vitamin A total body stores (TBS)]. In fact, in a recent publication, Gannon et al. (13) described retinol kinetic studies carried out for 97 to 152 d in 7 young women. When the authors analyzed serum retinol tracer data truncated from a median of 137 d to a median of 52 d, there was no significant reduction in predicted stores; however, when data were truncated to 14 d, predictions were significantly lower. Also, predictions of DR were significantly higher (7.44 compared with 2.95 µmol/d) using data truncated to 14 d than if full data sets were analyzed. In their article, the authors reinforce the point that longer studies may be required to accurately estimate vitamin A kinetic parameters and the size of slowly turning-over (storage) pools of vitamin A in adults.

In addition to study length and sampling protocol, there may be other ways to improve the identification of the terminal slope of the plasma retinol isotope response curve. Recently, a modeling challenge (specifically, a terminal slope that approached zero) that occurred during our work with López-Teros et al. in Mexican children (12) led us to the idea of using information on participants’ dietary vitamin A intake to constrain that slope to a non-zero value in the final steady state model. That is, because modeling, as it has been applied to the vitamin A system, assumes a steady state, constraining the value for input (dietary vitamin A intake × fractional absorption of vitamin A) also constrains the output. Reflecting back on the results of Cifelli et al. (10), we hypothesized that including an estimate of intake in the modeling data stream might provide the extra information needed to generate more realistic predictions of intake, DR, TBS, days of stores, and other kinetic parameters. Here, we reanalyzed the data from (17, 18) that were presented in (10) and demonstrate the advantage of including vitamin A intake data when modeling is used to estimate vitamin A TBS and DR in a kinetic study of less-than-optimal length. This approach enhances the power of model-based compartmental analysis for studying the kinetics of vitamin A and it can potentially be applied to other nutrients.

## Methods

### Subjects and kinetic studies

We used a subset of US and Chinese subjects from studies by Tang et al. (17) and Wang et al. (18), respectively. As described in detail in the original articles, subjects ingested an oral dose of [^2^H_8_]retinyl acetate. Serum retinol tracer was monitored from 3 h to 52 d and [^2^H_8_]retinol data versus time were modeled as described by Cifelli et al. (10). For the current work, we included subjects (7 US and 6 Chinese) whose serum [^2^H_8_]retinol tracer kinetics were identifiable from 20 d onwards.

### Model-based compartmental analysis

**FIGURE 1:**
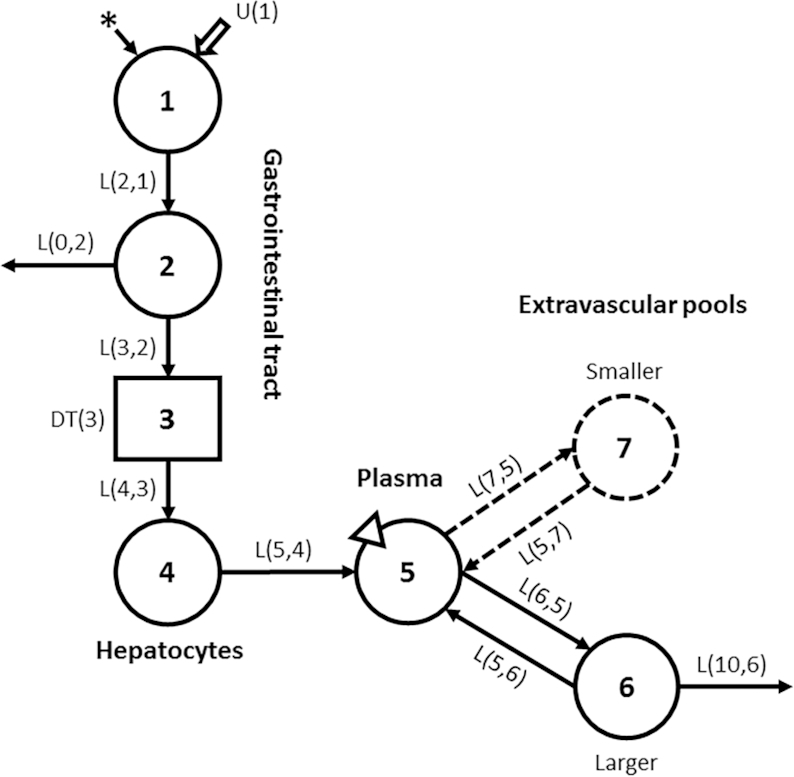
Compartmental model for vitamin A kinetics in humans. Circles represent compartments; the rectangle is a delay element, and DT(3) is the delay time spent in component 3; interconnectivities between components (arrows) are fractional transfer coefficients [L(I,J)s, or the fraction of retinol in compartment J transferred to compartment 1 each day]. Compartment 1 is the site of introduction of ingested tracer (*) and dietary vitamin A [U(1)]. Components 1–4 represent digestion, absorption, and chylomicron processing until uptake by hepatocytes (compartment 4), with subsequent secretion into plasma compartment 5 of retinol bound to retinol-binding protein; compartment 5 is the site of sampling (triangle). Retinol in plasma can exchange with vitamin A in 1 or 2 extravascular (EV) pools (a larger compartment 6, also the site of irreversible loss from the system, and a smaller compartment 7). Modeling was done with use of either the 6-compartment model (1 EV; components 1–6; solid lines) or the 7-compartment model (2 EV; components 1–7; solid and dashed lines).

To evaluate the usefulness of including an estimate of intake in the modeling data stream, we first analyzed mean data for the 2 groups as follows. We calculated the geometric mean [^2^H_8_]retinol fraction of dose in plasma (FD_p_) at each of the 19 sampling times from 3 h to 52 d for each group and fit the composite (population) data sets to (a) the 6-compartment model presented by Cifelli et al. (10), which included 1 extravascular compartment (1 EV), (b) a modified model that included 2 extravascular compartments (2 EV), and (c) the 2 EV model with an estimate of dietary vitamin A intake [2.8 µmol/d, the mean RDA for adult men and women (20)] included in the modeling data stream (2 EV DI) (**Figure 1**; see Results). Although our assumption was that the 1 EV model provided inaccurate estimates of intake (and thus of DR and TBS), we included it for comparison to the model used in the original analysis (10); note that the current work did not include all of the subjects presented in (10). As described in more detail in (10), current data were modeled using the Windows version of the Simulation, Analysis and Modeling software (version 3.3.0; www.WinSAAM.org) (2, 21). Model parameters [DT(I), delay time in component I, and L(I, J)s, fractional transfer coefficients or the fraction of retinol in compartment J that is transferred to compartment I each day] were adjusted to obtain a good fit of FD_p_ data (as well as intake in the case of 2 EV DI) to the models; final parameter values were obtained using weighted nonlinear regression analysis in WinSAAM, with a fractional SD of 0.05 as a weighting factor for plasma data and 0.1 for dietary data in the case of the 2 EV DI approach. Then, we calculated plasma retinol pool size (µmol) for each individual as described in (10) [mean of retinol concentrations (µmol/L) in the 19 serum samples × estimated plasma volume (L)] and used each group's geometric mean pool size in a steady state solution to obtain compartment masses, including TBS, and other state variables, including U(1), the input rate, and DR. Results were compared among the 3 approaches (1 EV, 2 EV, and 2 EV DI).

Following analysis of the population data sets, the modeling process was applied to each individual's data on FD_p_ compared with time. Mean results for individuals in each group were compared to those obtained by modeling the geometric mean data and additional kinetic parameters were calculated.

### Data manipulations and statistics

Results are presented as geometric means unless otherwise indicated. Data were managed with the use of Microsoft Excel and figures were created using GraphPad Prism 7.0 for Windows. Statistical analysis for subject demographics were done using a *t* test in JMP Pro 14; *P* < 0.05 was considered to be significant. Compartmental models were compared using an *F* statistic (22) to determine whether increasing model complexity (i.e., 1 EV compared with 2 EV or 2 EV DI) resulted in a significant improvement (*P* < 0.05) in the weighted sum of squares and was therefore statistically justified.

## Results

### Subject characteristics

Demographic characteristics, mean serum retinol concentrations, and estimated liver weights for the 13 subjects are shown in **Table 1**. The group of US subjects included 5 females and 2 males; half of the Chinese subjects (*n* = 3) were female. There were no significant differences between groups for any of the listed variables except that serum retinol concentrations, although within normal ranges for both groups, were significantly higher in US than Chinese participants [1.74 ± 0.364 compared with 1.20 ± 0.288 µmol/L (mean ± SD); *P* < 0.05]. Mean age for subjects included in the current analysis was 58 and 54 y (US and Chinese, respectively); corresponding body weights were 68 and 68 kg, BMIs were 26 and 27 kg/m^2^, and estimated liver weights, calculated based on body surface area (23), were 1.33 and 1.34 kg.

**TABLE 1:** Individual subject demographics, serum retinol concentrations, and liver weights^1^

ID	Sex, M/F	Age, y	Body weight, kg	BMI, kg/m^2^	[ROH], µmol/L	Liver weight, g
US1	M	47	72.6	29.3	2.34	1375
US2	F	55	57.7	25.7	1.64	1196
US3	F	44	82.3	30.7	1.44	1494
US4	F	70	54.5	22.0	1.41	1192
US5	F	57	60.4	27.9	2.06	1213
US6	M	68	79.5	25.9	1.41	1519
US7	F	67	65.8	26.6	1.85	1309
CH1	M	50	59.8	23.9	1.40	1251
CH2	M	60	63.8	21.6	1.35	1347
CH3	F	57	77.0	28.6	1.10	1446
CH4	F	51	56.9	23.7	0.932	1208
CH5	F	55	77.4	29.9	0.850	1436
CH6	M	53	72.0	30.0	1.58	1359

^1^Shown are subject characteristics for US (US1–7) and Chinese subjects (CH1–6), a subset of the individuals studied by Tang et al. (17) and Wang et al. (18), respectively. Retinol concentrations [ROH] are means based on analysis of the 19 serum samples collected from 3 h to 52 d after administration of [^2^H_8_]retinyl acetate, as described in (10). Estimated liver weights were calculated as body surface area × 772 (23).

### Compartmental models and model-derived parameters

As indicated in Figure 1, both the 1 EV and 2 EV models include 4 components (compartments 1, 2, and 4 plus delay element 3) that represent the processes of vitamin A digestion and absorption, chylomicron formation and catabolism, hepatic clearance of chylomicron remnants, and secretion of retinol bound to retinol-binding protein from the liver into plasma compartment 5 (retinol-binding protein-bound retinol). In the 6-compartment (1 EV) model presented by Cifelli et al. (10), retinol in compartment 5 exchanges with that in compartment 6, a large, slowly turning-over extravascular vitamin A storage pool which is the site of irreversible loss of vitamin A. In the 7-compartment (2 EV) model, there is an additional extravascular pool of vitamin A that exchanges with retinol in compartment 5: this compartment (compartment 7) is smaller than compartment 6. A 2 EV model was tested in (10) but it did not provide a significant improvement in the fit of the data based on the sum of squares of weighted residuals and an *F* statistic, and thus the simpler model was used. In the current analysis, 2 extravascular compartments [without (2 EV) or with (2 EV DI) dietary intake included in the data stream] were statistically justified to fit the population data sets (see subsequent sections).

**FIGURE 2:**
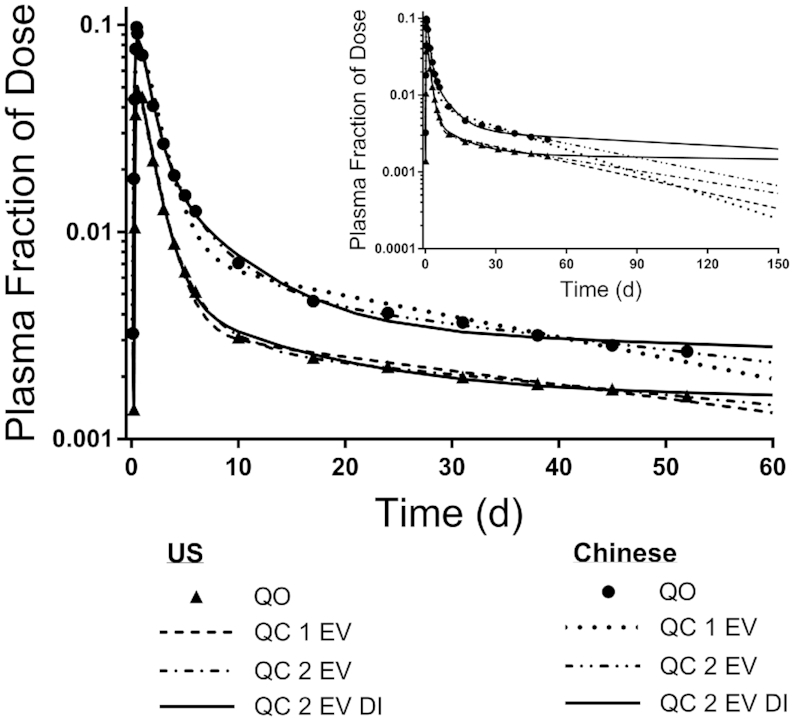
Model-predicted fraction of dose for [^2^H_8_]retinol in plasma versus time. Observed data (QO; symbols) are geometric mean fraction of dose for a group of older US (*n* = 7) and Chinese adults (*n* = 6) and model-calculated (QC) fits (lines) using a model (Figure 1) with 1 ‘extravascular’ compartment (1 EV) or 2 extravascular compartments (without or with ‘dietary intake’ data as an input; 2 EV and 2 EV DI, respectively). The inset shows model-simulated data to 150 d using the 3 models.

Geometric mean FD_p_ data and model fits using the 1 EV and 2 EV models (without and with dietary intake data included for 2 EV) for US and Chinese subjects are shown in **Figure 2** and final model parameters are presented in **Supplemental Table 1**; see **Supplemental WinSAAM Deck** for the 2 EV DI model applied to both groups. As shown in Figure 2, the curves for the US subjects peaked at a lower FD_p_ and remained lower over time, suggesting a higher TBS in that group, as noted by Cifelli et al. (10). When we simulated model-predicted data beyond the 52 d experimental period to 150 d, as shown in the inset to Figure 2, the difference in terminal slopes for 1 EV versus 2 EV versus 2 EV DI was even more evident. Overall, the use of the 2 EV and 2 EV DI (compared with 1 EV) models resulted in a statistically better fit of the data for both the US and Chinese groups (*P* < 0.01).

Steady state model solutions for the 1 EV, 2 EV, and 2 EV DI models based on analysis of the population data sets for US and Chinese subjects are presented in **Table 2**. Vitamin A intake rate predicted by the 2 EV model was lower than that predicted by the 1 EV model for both US (14 µmol/d compared with 16 µmol/d, respectively) and Chinese subjects (6 µmol/d compared with 7 µmol/d) but values were still much higher than the mean RDA of 2.8 µmol/d. That is, although the 2 EV model provided a better fit to the data for both groups, it did not affect the terminal slope of the curve enough to provide realistic predictions of dietary intake rate; consequently, we rejected that approach. However, when an estimate of vitamin A intake was included in the data stream (2 EV DI), the model-predicted intake rates for both groups were much lower (2.8 µmol/d and 2.9 µmol/d for US and Chinese, respectively) and close to the mean RDA, because of the constraining influence of the intake estimate on the terminal slope. The differences in predictions of intake rate by the 2 EV DI compared with 1 EV models were reflected in model-predicted disposal rates of 2.1 µmol/d compared with 12 µmol/d for US subjects [reflecting a difference in fractional loss from compartment 6 (Figure 1) of 0.11%/d compared with 1.6%/d; Supplemental Table 1] and 2.2 µmol/d compared with 5.1 µmol/d for the Chinese (reflecting a difference of 0.39%/d compared with 2.3%/d). For the US subjects, calculated mean TBS (Table 2) was 2056 µmol compared with 783 µmol for the 2 EV DI compared with 1 EV models (a 1.6-fold increase) and 594 µmol compared with 219 µmol for the Chinese subjects (a 1.7-fold increase); these values translate to liver vitamin A concentrations of 1.4 µmol/g compared with 0.53 µmol/g (US; 2 EV DI compared with 1 EV) and 0.40 µmol/g compared with 0.15 µmol/g (Chinese). Estimated number of days of stores provided by these amounts of TBS were 981 d compared with 64 d for the US subjects (2 EV DI compared with 1 EV), a 14-fold increase, and 269 d compared with 43 d for Chinese, representing a 5.2-fold increase. Note that, although values predicted here by the 1 EV model are slightly different from those reported in (10) because of remodeling and the subjects selected for this analysis, relative conclusions are the same. Specifically, predictions of TBS and days of stores were higher in the US compared with Chinese groups.

**TABLE 2:** Model predictions and estimated liver vitamin A for population data sets^1^

	US			Chinese		
	1 EV	2 EV	2 EV DI	1 EV	2 EV	2 EV DI
Dietary intake [U(1), µmol/d]	16.2	14.4	2.79	6.84	5.86	2.94
Plasma retinol pool [M(5), µmol]	5	5	5	3.43	3.43	3.43
Extravascular vitamin A pools, µmol						
M(6)	783	926	1926	219	297	564
M(7)	NA	29.7	130	NA	19.6	30.1
TBS	783	956	2056	219	317	594
DR, µmol/d	12.2	10.8	2.1	5.13	4.39	2.21
Days of stores, d	64.4	88.5	981	42.8	72.1	269
Liver vitamin A, µmol/g	0.533	0.65	1.4	0.148	0.213	0.4

^1^Results are model-predicted values obtained using a model (Figure 1) with 1 extravascular pool (1 EV) or 2 extravascular pools (without or with dietary intake as an input; 2 EV and 2 EV DI, respectively) using geometric mean plasma tracer response data for a group of US (*n* = 7) and Chinese subjects (*n* = 6). Values, calculated using the geometric mean plasma retinol pool size [M(5)] in a steady state solution, include dietary vitamin A intake, mass of vitamin A in compartments 6 and 7 and in TBS, and vitamin A disposal rate [calculated as M(6) × L(10,6) (see Supplemental Table 1 and Figure 1)]; TBS for the 1 EV model equals M(6) and for the 2 EV and 2 EV DI models, TBS equals M(6) + M(7). Also shown are days of vitamin A stores, calculated as TBS/DR, and liver vitamin A concentrations, calculated as (model-predicted TBS × 0.9)/geometric mean liver weight in grams (Table 1), using the assumption that 90% of total body vitamin A is found in the liver (24). DR, disposal rate; M(I), mass of vitamin A in compartment I; TBS, total body stores.

Kinetic parameters obtained by modeling tracer response data for individual subjects using the 2 EV DI model are presented, along with values predicted for the geometric mean data sets, in Supplemental Table 1. Overall, the geometric mean of the individual model-predicted values for kinetic parameters for the 2 groups agreed well with those calculated for the population data sets. Note that, when individual subjects’ data were fit to the 1 EV and 2 EV models (without or with diet constraint for the latter), predictions of kinetic parameters followed similar trends to those observed for the geometric mean data sets (data not shown). As shown in **Supplemental Table 2**, the model-predicted values for TBS for individual subjects were consistently higher using the 2 EV DI compared to the 1 EV model; the difference was greater for the US individuals (ranging from a 0.69 to a 6.6-fold increase) than for the Chinese (a 0.54 to a 2.3-fold increase). In addition, using the 2 EV DI model, the geometric mean of the individual model-calculated values for TBS was within 9% of the value predicted for the population data sets for the US group and within 2% of that value for the Chinese group. Results for model-predicted intake and disposal rates as well as calculated liver vitamin A concentrations and days of stores for individuals followed similar trends to those observed for the population data sets (Supplemental Table 2). Individual and geometric mean values for additional kinetic parameters are presented in **Supplemental Table 3**, providing information on vitamin A transit times, residence times, and recycling times and numbers in US and Chinese well-nourished older adults.

## Discussion

As noted in our Introduction, it is known (16) that study duration impacts both model structure and parameter predictions when compartmental analysis is applied to the vitamin A system. For example, if an experiment is not carried out for long enough to define the true terminal slope of the isotope response curve, DR will be overestimated because the apparent slope is steeper than the true terminal slope. Under the assumption of a steady state, this results in a concomitant overestimation of vitamin A intake and an underestimation of TBS. Especially when vitamin A stores are high, very long studies (∼400 d) may be required to accurately define the final slope of the curve (25). Indeed, when investigators in Tanumihardjo's laboratory recently evaluated the influence of study duration on predictions of TBS and retinol kinetics in women using compartmental modeling (13), they concluded that, as the duration of the experiment increased, predictions of DR decreased and estimates of total traced mass (primarily vitamin A stores) increased. In addition, based on the results of a very long-term study (6 y) in 1 subject, they suggested that a duration of >200 d may be required to accurately estimate parameters of interest; this agrees with the earlier recommendation made by Green and Green (15).

In retrospect, it seems clear that a less-than-optimal study length led to the unrealistic estimates of DR reported by Cifelli et al. (10) for older, well-nourished US and Chinese adults. By including an estimate of vitamin A intake as an additional modeling input in the current analysis, as originally done in our work with López-Teros et al. (12) and more recently in a theoretical analysis (26), we obtained revised estimates of vitamin A TBS and DR. Specifically, inclusion of the mean RDA for vitamin A (2.8 µmol/d) as an estimate of intake resulted in predictions (Table 2) of 2.8 µmol/d (US) and 2.9 µmol/d (Chinese) for vitamin A intake, values that are much more realistic given the experimental conditions than the values reported in (10) (15 and 6 µmol/d, respectively). Updated predictions of DR were also lower (∼2 µmol/d for both groups or 83 and 57% lower for US and Chinese subjects, respectively), whereas predictions of TBS were around 3 times higher than those presented in (10). Accurate prediction of TBS is important because compartmental analysis is now recognized as an additional way to assess vitamin A status (27).

It is also worth noting that our updated estimate of liver vitamin A concentration (1.4 µmol/g for the US subjects) is higher than the cutoff of 1 µmol/g that has been suggested for hypervitaminosis A (28). However, no signs or symptoms of vitamin A excess or toxicity were reported in the original studies by Tang et al. (17) and Wang et al. (18).

In our current analysis, we used the mean RDA as an estimate of intake because vitamin A intake data from the original studies (17, 18) were not available. To investigate the impact of this choice, we also evaluated inputs of 50% and 150% of the RDA (i.e., 1.4 and 4.2 µmol/d) and found that predicted TBS changed by a maximum of 8% for the US subjects and 28% for the Chinese. Although this suggests that a reasonable estimate of intake may be adequate, it would be ideal to measure participants’ vitamin A intake data for inclusion in future modeling analyses, given its potential impact on modeling results.

In the work of Cifelli et al. (10), a 6-compartment model that included 1 extravascular pool (Figure 1) provided the best fit to plasma tracer response data over a 52 d experiment. When we added estimates for intake into the modeling data stream, a second, smaller extravascular pool was required to fit the data. A second extravascular pool has been included in some previous vitamin A models in humans (12, 13, 15, 29) but not in others (10, 11). Whether or not a second pool is identified depends on the study length and sampling schedule. Although the absolute value of model predictions of TBS and DR differed when using the 1 EV model presented by Cifelli et al. (10) and the 2 EV model with an estimate of intake included (2 EV DI), the results were qualitatively similar between groups. Specifically, predictions of vitamin A stores and DR were higher in US than Chinese subjects.

In conclusion, this work describes a promising step forward in compartmental modeling of vitamin A kinetics: specifically, that including reasonable estimates for dietary vitamin A intake as an input during the modeling process leads to realistic predictions of TBS, days of vitamin A stores, and DR from kinetic studies of relatively short duration (10). As a next step, it will be important for investigators to evaluate the accuracy of model predictions when intake data are included by using theoretical data and known values for TBS and kinetic parameters to test the approach, as we have recently done to evaluate other methods (26, 30, 31). It is anticipated that such work will confirm our conclusion that including vitamin A intake data as an additional input when modeling vitamin A kinetics can improve predictions of vitamin A TBS and DR by compensating for less-than-optimal study duration. Such an approach represents an advance in the application of compartmental analysis to the study of nutrient kinetics, potentially extending beyond the vitamin A system to the modeling of other nutrients.

## Supplementary Material

nxz056_Supplemental_File

## Notes

Support for this work was provided by the Bill & Melinda Gates Foundation (Project Number OPP1115464) and the College of Health and Human Development, The Pennsylvania State University.

Supplemental Tables 1–3, Supplemental WinSAAM Deck, and Supplemental References are available from the “Supplementary data” link in the online posting of the article and from the same link in the online table of contents at https://academic.oup.com/jn/.

MHG and JLF contributed equally to this work.

Author disclosures: MHG, JLF, and JBG, no conflicts of interest.

Abbreviations used: DI, dietary intake; DR, disposal rate; EV, extravascular; FD_p_, fraction of administered dose in plasma; TBS, total body stores.
