# Supplementary material for: Inclusion of Vitamin A Intake Data Provides Improved Compartmental Model-Derived Estimates of Vitamin A Total Body Stores and Disposal Rate in Older Adults
Source: J Nutr. 2019 May 16;149(7):1282–7. doi: 10.1093/jn/nxz056 (PMC6602889; doi:10.1093/jn/nxz056)
Supplement: nxz056_Supplemental_File [file nxz056_supplemental_file.pdf]

Supplemental Table 1 Kinetic parameters for geometric mean models and individual subjects[1]

Parameter by group	Population (GM) models			Individual fit to 2 EV DI model							
US	1 EV	2 EV	2 EV DI	US1	US2	US3	US4	US5	US6	US7	GM
L(2,1), d^{-1}	50	50	50	50	50	50	50	50	50	50	50
L(5,2), d^{-1}	0.355	0.460	0.386	0.526	1.64	0.370	1.02	1.10	0.229	0.370	0.608
DT(3), d	0.237	0.243	0.239	0.230	0.251	0.229	0.263	0.236	0.251	0.235	0.242
L(5,4), d^{-1}	0.658	0.806	0.714	0.754	0.600	0.693	0.816	1.88	0.639	0.857	0.827
MST_{RBP}, d	1.80	1.52	1.68	1.60	1.96	1.71	1.53	0.806	1.86	1.44	1.51
L(0,2), d^{-1}	16.8	16.8	16.8	16.8	17.2	16.8	17.0	17.0	16.7	16.8	16.9
L(6,5), d^{-1}	7.85	7.25	5.29	3.48	6.18	4.00	18.0	4.28	2.24	6.02	5.10
L(5,6), d^{-1}	0.0346	0.0275	0.0126	0.0102	0.0640	0.00810	0.0212	0.0320	0.00355	0.0181	0.0156
L(10,6), d^{-1}	0.0155	0.0117	0.00109	0.000906	0.000438	0.000878	0.000769	0.00514	0.000762	0.00141	0.00106
L(7,5), d^{-1}		2.28	3.09	2.05	14.7	3.71	6.75	6.69	2.58	2.28	4.36
L(5,7), d^{-1}		0.383	0.119	0.191	0.0121	0.100	0.292	1.16	0.0814	0.156	0.139
Chinese	1 EV	2 EV	2 EV DI	CH1	CH2	CH3	CH4	CH5	CH6		GM
L(2,1), d^{-1}	50	50	50	50	50	50	50	50	50		50
L(5,2), d^{-1}	0.315	0.360	0.346		0.300	0.0109		0.186	1.32		0.168
DT(3), d	0.136	0.151	0.150	0.0689	0.155	0.249	0.0787	0.146	0.161		0.130
L(5,4), d^{-1}	0.638	0.906	0.861	1.50	0.589	1.17	0.923	0.868	1.54		1.04
MST_{RBP}, d	1.74	1.30	1.35	0.776	1.89	1.14	1.20	1.34	0.849		1.15
L(0,2), d^{-1}	16.8	16.8	16.8	16.6	16.7	16.6	16.6	16.7	17.1		16.8
L(6,5), d^{-1}	4.09	3.30	2.86	2.96	3.92	3.31	3.75	2.83	1.38		2.87
L(5,6), d^{-1}	0.0407	0.0234	0.0135	0.0165	0.00834	0.0152	0.0136	0.0154	0.0156		0.0138
L(10,6), d^{-1}	0.0234	0.0148	0.00392	0.00492	0.00141	0.00325	0.00468	0.00557	0.00732		0.00403
L(7,5), d^{-1}		2.32	2.39	3.87	2.10	3.034	3.29	2.042	4.92		3.06
L(5,7), d^{-1}		0.407	0.272	0.699	0.0921	0.426	0.262	0.477	0.588		0.356

[1] Values are kinetic parameters calculated using a model (Figure 1) with either 1 extravascular pool (1 EV) or 2 extravascular pools (without or with dietary intake as an input; 2 EV and 2 EV DI, respectively) for geometric mean datasets for a group of US (US1 – 7) and Chinese (CH1 – 6) subjects. In all cases, we fixed L(2,1) (Figure 1), representing movement down the GI tract, at 50/d based on previous work in this lab on these same subjects (**Supplemental Reference 1**). Also shown are kinetic parameters calculated for individuals using the 2 EV DI model. Kinetic parameters are L(I,J)s, or the fraction of retinol in compartment J transferred to compartment I each day; DT(3), or the time (d) spent in delay component 3 (Figure 1); and MST_{RBP}, mean sojourn time to retinol-binding protein, calculated as the sum of the turnover times for compartments 1, 2 and 4 plus the time (d) in delay component 3, or [1 / L(2,1) + 1 / L(3,2) + 1 / L(5,4) + DT(3)] × 24, where L(3,2) = L(2,1). See **Supplemental Table 3** for additional calculated parameters that correspond to the results presented here. GM, geometric mean.

Supplemental Table 2 Steady state model predictions, days of vitamin A stores and estimated liver vitamin A concentrations for individual subjects[1]

ID	U(1) 1 EV	U(1) 2 EV DI	DR 1 EV	DR 2 EV DI	M(5)	M(6) 1EV	M(6) 2 EV DI	M(7) 2 EV DI	TBS 2 EV DI	Days of stores 1 EV	Days of stores 2 EV DI	Liver VA 1 EV	Liver VA 2 EV DI
US1	19.2	2.79	14.4	2.10	7.41	675	2312	79.3	2391	46.9	1141	0.441	1.56
US2	27.9	2.81	20.9	2.10	4.11	2669	4810	397	5206	127.7	2473	2.01	3.92
US3	18.3	2.82	13.7	2.12	5.41	649	2409	201	2610	47.3	1233	0.391	1.57
US4	20.1	2.82	15.1	2.11	3.34	1668	2745	77.3	2823	110	1337	1.26	2.13
US5	6.34	3.21	4.76	3.21	5.42	293	625	31.3	656	61.6	204	0.217	0.487
US6	12.8	2.80	9.64	2.10	5.30	385	2753	168	2922	40.0	1393	0.265	2.01
US7	13.2	2.82	9.94	2.11	4.86	781	1503	71.2	1574	78.7	745	0.463	0.933
**GM**	**15.5**	**2.86**	**11.6**	**2.24**	**4.99**	**778**	**2116**	**109**	**2233**	**67.0**	**998**	**0.529**	**1.52**
CH1	7.64	3.30	5.73	2.10	3.65	168	502	20.2	523	29.2	211	0.120	0.376
CH2	11.4	2.81	8.54	2.10	3.73	477	1500	85.2	1586	55.8	752	0.319	1.06
CH3	6.04	2.85	4.53	2.12	3.67	444	658	26.1	684	98.1	320	0.277	0.426
CH4	6.16	2.95	4.62	2.11	2.31	181	472	29.0	501	39.2	227	0.135	0.373
CH5	4.80	2.87	3.60	3.21	2.86	223	386	12.2	398	62.0	185	0.140	0.250
CH6	5.32	2.91	3.99	2.10	4.96	102	298	41.4	340	25.5	156	0.067	0.225
**GM**	**6.60**	**2.94**	**4.95**	**2.26**	**3.43**	**230**	**547**	**29.5**	**581**	**46.3**	**263**	**0.154**	**0.391**

[1]Results are model-predicted values obtained using a model (Figure 1) with 1 extravascular pool (1 EV) or 2 extravascular pools (without or with dietary intake as an input; 2 EV and 2 EV DI, respectively) for US (US1 – 7) and Chinese (CH1 – 6) subjects. Shown are values calculated using the plasma retinol pool size [M(5), µmol] in a steady state solution; values include dietary vitamin A intake [U(1), µmol/d], mass of vitamin A in compartments 6 and 7 and in TBS (µmol), and vitamin A disposal rate [DR, µmol/d, calculated as M(6) × L(10,6) (see Supplemental Table 1)]; TBS for the 1 EV model equals M(6) and for the 2 EV and 2 EV DI models, TBS equals M(6) + M(7). Also shown are days of vitamin A stores (d), calculated as TBS / DR, and liver vitamin A concentrations (µmol/g), calculated as model-predicted TBS / liver weight (g, see Table 1). DR, disposal rate; GM, geometric mean; M(I), mass of vitamin A in compartment I; TBS, total body stores.

Supplemental Table 3 Additional calculated kinetic parameters for individual subjects[1]

Parameter by group	Population (GM) models			Individual fit to 2 EV DI model							
US	1 EV	2 EV	2 EV DI	US1	US2	US3	US4	US5	US6	US7	GM
$\bar{t}$(5), d	0.127	0.105	0.119	0.181	0.0479	0.130	0.0404	0.0912	0.207	0.120	0.100
$\bar{t}$(6), d	28.9	36.4	79.1	97.6	15.6	124	47.2	31.2	282	55.3	63.9
$\bar{t}$(7), d		2.61	8.39	5.22	82.3	10.0	3.42	0.864	12.3	6.41	7.20
$\bar{T}$(5,5), d	0.411	0.463	2.39	3.54	1.95	2.55	1.58	1.69	2.52	2.30	2.23
$\bar{T}$(6,5), d	64.4	85.7	919	1104	2285	1138	1300	195	1313	711	946
$\bar{T}$(7,5), d		2.75	61.9	37.8	188	94.9	36.6	9.75	80.2	33.7	48.7
$\bar{T}$(SYS), d	64.8	89.0	984	1145	2475	1236	1338	206	1395	747	1001
ν(5)	2.23	3.41	19.0	18.5	39.8	18.7	38.2	17.5	11.2	18.1	21.1
$\overline{tt}$(5), d	28.9	25.95	51.6	61.5	62.1	66.0	35.0	11.7	124	41.1	47.2
Chinese	1 EV	2 EV	2 EV DI	CH1	CH2	CH3	CH4	CH5	CH6		GM
$\bar{t}$(5), d	0.244	0.178	0.190	0.146	0.166	0.158	0.142	0.205	0.159		0.161
$\bar{t}$(6), d	24.6	42.8	74.0	60.4	120	65.6	73.4	64.8	64.0		72.4
$\bar{t}$(7), d		2.46	3.67	1.43	10.8	2.34	3.82	2.09	1.70		2.81
$\bar{T}$(5,5), d	0.670	0.781	1.55	1.48	1.77	1.72	1.04	1.33	2.27		1.56
$\bar{T}$(6,5), d	42.8	67.7	255	203	711	308	214	180	137		248
$\bar{T}$(7,5), d		4.455	13.6	8.16	40.4	12.2	13.1	5.69	19.0		13.4
$\bar{T}$(SYS), d	43.5	72.9	271	213	753	322	228	186	157.9		265
ν(5)	1.74	3.39	7.17	9.07	9.65	9.90	6.35	5.49	13.30		8.59
$\overline{tt}$(5), d	24.6	21.2	37.5	23.3	77.9	32.3	35.7	33.8	11.7		30.6

[1]Values are kinetic parameters calculated using results from a model (Figure 1) with either 1 extravascular pool (1 EV) or 2 extravascular pools (without or with dietary intake as an input; 2 EV and 2 EV DI, respectively) for geometric mean datasets for a group of US (US1 – 7) and Chinese (CH1 – 6) subjects. Calculated parameters include: transit times [$\bar{t}$(J), or the mean of the distribution of times a retinol molecule spends in compartment J during a single transit, calculated as 1/∑L(I,J)s exiting compartment J] for retinol in plasma [$\bar{t}$(5)] and extravascular compartments 6 [$\bar{t}$(6)] and 7 [$\bar{t}$(7)]; mean residence times [$\bar{T}$(I,J), or the mean of the distribution of times the tracer spends in compartment I after entering the system via compartment J, where J is compartment 5 for tracer that was absorbed] for retinol in plasma [$\bar{T}$(5,5)], the extravascular compartments 6 and 7 [$\bar{T}$(6,5) and $\bar{T}$(7,5), respectively], and the system [$\bar{T}$(SYS), equal to $\bar{T}$(5,5) + $\bar{T}$(6,5) + $\bar{T}$(7,5)]; recycling number for plasma {ν(5), or the mean number of times a molecule of retinol recycles to plasma before being irreversibly lost, calculated as [$\bar{T}$(5,5) / $\bar{t}$(5)] – 1}; and recycling time to plasma {$\overline{tt}$(5), calculated as [$\bar{T}$(6,5) + $\bar{T}$(7,5)] / ν(5)}. For more information on calculation of parameters, see (3). GM, geometric mean.

Supplemental WinSAAM Deck

```
A SAAM31            US & CH POPULATION DATASETS [12-DEC-2018]
CC 7-COMPARTMENT MODEL FOR VITAMIN A
CC INCLUDING 2 EXTRAVASCULAR (EV) COMPARTMENTS AND DIETARY INTAKE (2 EV DI)
CC US POPULATION (GEMEOTRIC MEAN) DATASET (N=7)
CC PARAMETERS
H PAR
CC          VALUE          LOWER LIMIT    UPPER LIMIT
CC IC(I)=INITIAL CONDITION (FRACTION OF DOSE) IN COMPARTMENT I AT TIME 0
   IC(1)=1.0
CC L(I,J)=FRACTION OF J TRANSFERRED TO I PER DAY (DAY^-1)
   L(2,1)    50
   L(3,2)=L(2,1)
   L(5,2)    3.859802E-01  0.000000E+00   5.000000E+00
CC L(0,2)=FRACTIONAL LOSS OF UNABSORBED TRACER
CC ASSUMING 75% ABSORPTION EFFICIENCY; L(0,2)=1/3 OF [L(2,1)+L(5,2)]
   L(0,2)=0.333*(L(3,2)+ L(5,2))
CC DN(I)=NUMBER OF ELEMENTS IN DELAY COMPONENT I
CC DT(I)=DELAY TIME IN COMPONENT I (DAY)
   DN(3)     8
   DT(3)     2.394382E-01  0.000000E+00   1.000000E+00
CC OUTPUT FROM DELAY COMPONENT EQUALS 1
   L(4,3)=1.0
   L(5,4)    7.138983E-01  0.000000E+00   1.000000E+01
   L(6,5)    5.287296E+00  0.000000E+00   2.500000E+01
   L(5,6)    1.263508E-02  0.000000E+00   2.000000E+01
CC L(10,6)=FRACTIONAL CATABOLIC RATE OF COMPARTMENT 6;
CC COMPARTMENT 10 IS LOSS FROM THE SYSTEM
   L(10,6)   1.087773E-03  0.000000E+00   1.000000E+00
   L(7,5)    3.094179E+00  0.000000E+00   2.500000E+01
   L(5,7)    1.192151E-01  0.000000E+00   1.000000E+01
CC STEADY STATE SOLUTION
CC U(I)=VITAMIN A INTAKE RATE (UMOL/D)
CC M(I)=MASS OF VITAMIN A IN COMPARTMENT I (UMOL)
H STE
   U(1)     2.793367       0.0            100
CC M(5)=PLASMA RETINOL POOL (UMOL)
   M(5)     5
CC MODEL-PREDICTED MASSES IN COMPARTMENTS 6 & 7
CC M(6)=1926 UMOL
CC M(7)=130  UMOL
CC DATA
H DAT
CC WEIGHTED U(1) AT AVERAGE RDA OF 2.8 UMOL/D
CC          TIME (D)      U(1)           FRACTIONAL STANDARD DEVIATION
100                                      FSD=0.10
   U(1)     0.0           2.8
CC PLASMA RETINOL FROM ORAL [D8]RETINYL ACETATE
105                                      FSD=0.05
CC GEOMETRIC MEAN OF OBSERVED (QO) DATA FOR 7 US SUBJECTS AT ALL TIMES
CC EXCEPT 2 EV MODEL-CALCULATED (QC) DATA USED FOR US3 AT 7 TIMES FROM
CC 10 TO 52D WHEN TIMES DIFFERED; 4 OUTLIERS (US1 AT 38D, US2 AT 31D,
CC US4 AT 17D, US7 AT 17D); 2 MISSING (US1 AT 45D, US3 AT 13HR)
```

```
CC          TIME (D)      PLASMA FRACTION OF ORAL DOSE
            0             0
            0.125         0
            0.2083        0.001390934
            0.2917        0.010563772
            0.375         0.03713231
            0.4583        0.04772708
            0.5417        0.046686674
            1             0.044926003
            2             0.022206398
            3             0.012916192
            4             0.008823351
            5             0.006459032
            6             0.005154786
            10            0.003098615
            17            0.002476407
            24            0.002240187
            31            0.00199649
            38            0.001850071
            45            0.001741402
            52            0.001611746
CC CHINESE POPULATION (GEOMETRIC MEAN) DATASET (N=6)
CC PARAMETERS
H PAR
CC          VALUE         LOWER LIMIT    UPPER LIMIT
CC IC(I)=INITIAL CONDITION (FRACTION OF DOSE) IN COMPARTMENT I AT TIME 0
   IC(11)=1.0
CC L(I,J)=FRACTION OF J TRANSFERRED TO I PER DAY (DAY^-1)
   L(12,11) 50
   L(13,12)=L(12,11)
   L(15,12) 3.495518E-01  0.000000E+00   5.000000E+00
CC L(0,12)=FRACTIONAL LOSS OF UNABSORBED TRACER
CC ASSUMING 75% ABSORPTION EFFICIENCY; L(0,12)=1/3 OF [L(12,11)+L(15,12)]
   L(0,12)=0.333*(L(13,12)+ L(15,12))
CC DN(I)=NUMBER OF ELEMENTS IN DELAY COMPONENT I
CC DT(I)=DELAY TIME IN COMPONENT I (DAY)
   DN(13)   8
   DT(13)   1.500189E-01  0.000000E+00   1.000000E+00
   L(14,13)=1.0
   L(15,14) 8.614130E-01  0.000000E+00   1.000000E+01
   L(16,15) 2.865249E+00  0.000000E+00   2.500000E+01
   L(15,16) 1.352236E-02  0.000000E+00   2.000000E+01
CC L(20,16)=FRACTIONAL CATABOLIC RATE OF COMPARTMENT 16;
CC COMPARTMENT 20 IS LOSS FROM THE SYSTEM
   L(20,16) 3.915200E-03  0.000000E+00   1.000000E+00
   L(17,15) 2.388299E+00  0.000000E+00   2.500000E+01
   L(15,17) 2.721264E-01  0.000000E+00   1.000000E+01
CC STEADY STATE SOLUTION
CC U(I)=VITAMIN A INTAKE RATE (UMOL/D)
CC M(I)=MASS OF VITAMIN A IN COMPARTMENT I (UMOL)
H STE
   U(11)    2.943978      0.0            100
CC M(5)=PLASMA RETINOL POOL (UMOL)
   M(15)    3.433
```

```
CC MODEL-PREDICTED MASSES IN COMPARTMENTS 16 & 17
CC M(16)=564 UMOL
CC M(17)=30.1 UMOL
CC DATA
H DAT
CC WEIGHTED U(11) AT AVERAGE RDA OF 2.8 UMOL/D
CC          TIME (D)     U(11)          FRACTIONAL STANDARD DEVIATION
100                                     FSD=0.10
   U(11)    0.0          2.8
CC PLASMA RETINOL FROM ORAL [D8]RETINYL ACETATE
115                                     FSD=0.05
CC GEOMETRIC MEAN OF OBSERVED (QO) DATA FOR 6 CHINESE SUBJECTS AT ALL
CC TIMES EXCEPT 2 EV MODEL-CALCULATED (QC) DATA USED FOR 1 OUTLIER
CC (CH1 AT 52D)
CC          TIME (D)      PLASMA FRACTION OF ORAL DOSE
            0             0
            0.125         0.003235811
            0.2083        0.018085579
            0.2917        0.043738916
            0.375         0.076390024
            0.4583        0.097550717
            0.5417        0.09094774
            1             0.07137227
            2             0.040691118
            3             0.026686389
            4             0.018733021
            5             0.015039936
            6             0.012597933
            10            0.007081779
            17            0.004640625
            24            0.004063006
            31            0.003654937
            38            0.003173681
            45            0.002839414
            52            0.002647928
```

Supplemental Reference

1. Park H, Green MH. Parameter identifiability and Extended Multiple Studies Analysis of a compartmental model for human vitamin A kinetics: fixing fractional transfer coefficients for the initial steps in the absorptive process. Br J Nutr 2014;111:1004-10.
